# Circulating microRNAs in young individuals with long-duration type 1 diabetes in comparison with healthy controls

**DOI:** 10.1038/s41598-023-38615-7

**Published:** 2023-07-19

**Authors:** Diana Swolin-Eide, Gun Forsander, Auste Pundziute Lyckå, Daniel Novak, Johannes Grillari, Andreas B. Diendorfer, Matthias Hackl, Per Magnusson

**Affiliations:** 1 Department of Pediatrics, Institute for Clinical Sciences, Sahlgrenska Academy, University of Gothenburg, Gothenburg, Sweden; 2 Department of Pediatrics, Region Västra Götaland, Sahlgrenska University Hospital, Queen Silvia Children’s Hospital, Gothenburg, Sweden; 3 Ludwig Boltzmann Institute for Traumatology, the Research Center in Cooperation With AUVA, Vienna, Austria; 4 Institute of Molecular Biotechnology, BOKU – University of Natural Resources and Life Sciences, Vienna, Austria; 5 Austrian Cluster for Tissue Regeneration, Vienna, Austria; 6 TAmiRNA GmbH, 1110 Vienna, Austria; 7 Department of Clinical Chemistry, and Department of Biomedical and Clinical Sciences, Linköping University, 581 85 Linköping, Sweden

**Keywords:** Diabetes, Biomarkers

## Abstract

MicroRNAs (miRNAs) are short non-coding RNAs that are involved in post-transcriptional control of gene expression and might be used as biomarkers for diabetes-related complications. The aim of this case–control study was to explore potential differences in circulating miRNAs in young individuals with long-duration type 1 diabetes (T1D) compared to healthy controls, and how identified miRNAs are expressed across different tissues. Twelve adolescents, age 15.0–17.9 years, with T1D duration of more than 8 years (mean 11.1 years), were enrolled from the Swedish diabetes quality registry. An age-matched control group was recruited. Circulating miRNAs (n = 187) were analyzed by quantitative PCR. We observed that 27 miRNAs were upregulated and one was downregulated in T1D. Six of these miRNAs were tissue-enriched (blood cells, gastrointestinal, nerve, and thyroid tissues). Six miRNAs with the largest difference in plasma, five up-regulated (hsa-miR-101-3p, hsa-miR-135a-5p, hsa-miR-143-3p, hsa-miR-223-3p and hsa-miR-410-3p (novel for T1D)) and one down-regulated (hsa-miR-495-3p), with *P*-values below 0.01, were selected for further in-silico analyses. AKT1, VEGFA and IGF-1 were identified as common targets. In conclusion, 28 of the investigated miRNAs were differently regulated in long-duration T1D in comparison with controls. Several associations with cancer were found for the six miRNAs with the largest difference in plasma.

## Introduction

Diabetes mellitus has become a global public health issue since more than 463 million people worldwide have diabetes and this number is projected to reach 578 million by 2030^1^. The evidence is clear that the degree of metabolic control can predict cardiovascular disease and premature death^2^. Prevention is the most effective way to reduce the occurrence of diabetes-related complications and all-cause mortality. Biomarkers, as early predictors for individual increased risk for type 1 diabetes (T1D) and for development of cardiovascular and other organ complications are of interest in targeted diabetes interventions.

MicroRNAs (miRNAs) are short non-coding RNAs that partake in the post-transcription control of gene expression. To date, approximately 2,600 human miRNAs have been identified from publicly available small RNA-sequencing data sets^3^. Following the transcription of long primary miRNA transcripts (> 200nt), sequential processing in the nucleus and cytoplasm occurs and yields mature miRNA duplexes of approximately 21–24 nucleotides in length^4^. In the cytoplasm, miRNA duplexes are separated, and single-stranded mature miRNAs are loaded into the RNA-induced silencing complex. Mature miRNA acts as a guide to direct the RNA-induced silencing complex to a target messenger RNA that contains one or several complementary sequences in its 3′ UTR. This interaction results in post-transcriptional silencing called RNA interference.

In 2008, the presence of miRNAs outside cells was confirmed in human plasma samples^5^. It is now clear that miRNAs can exit cells via active (transport) or passive (cell death) release mechanisms, and that the association of miRNAs to protein complexes or extracellular vesicles protects them from degradation^6^. Pre-analytical variation due to collection time-point, collection protocol, sample processing, storage, analytical variation due to the measurement protocol, biological variation derived from ethnical differences, lifestyle and comorbidities, can impact miRNA measurements and mask disease-related effects^7^. The presence of miRNAs in biofluids, the good measurability via techniques such as reverse transcription quantitative PCR (RT-qPCR) and next-generation sequencing, the tissue-specificity (approximately 5% of miRNAs), and their disease-relevance have established circulating miRNAs as promising biomarker candidates for both acute and chronic diseases^7^.

There are some reports on circulating miRNA levels in children with T1D^8^. In children with new-onset T1D, twelve miRNAs were found to be up-regulated in serum and associated with apoptosis and beta-cell function, including hsa-miR-25, which is predominately transcribed in the vasculature^9^. Åkerman et al.^10^ found that children with recently diagnosed T1D displayed highly altered circulating miRNA profiles, while no specific miRNA profiles were identified in individuals at high-risk for T1D.

Circulating miRNAs have been identified as potential biomarkers for long-term diabetes-related complications in T1D patients. To our knowledge, no study has thus far investigated circulating miRNA levels in children with long-duration T1D. This case–control discovery study was designed to explore if there are any differences in circulating miRNAs between Swedish adolescents with long-duration T1D and healthy control subjects. In addition, we also investigated how the identified miRNAs are expressed across tissues, which could be of potential interest for the prognosis of diabetes-related long-term complications.

## Materials and methods

### Subjects and study design

Twelve adolescents aged 15.0–17.9 years, 7 males and 5 females, with a long-term T1D duration of at least 8 years (8.0–15.0 years) were included in this case–control study. These individuals were followed regularly at the Queen Silvia Children’s Hospital in Gothenburg, Sweden. They were identified from the Swedish Pediatric Diabetes Quality Registry (SWEDIABKIDS). Since 2007, all 43 paediatric clinics in Sweden prospectively report clinical follow-up visit data for children with diabetes to SWEDIABKIDS. This registry is funded by the Swedish Association of Local Authorities and Regions. It has been web-based since 2008 and is estimated to include 97.5% of children aged 0–17.99 years.

The control group comprised 12 age-matched healthy adolescents, 15.0–17.5 years, 6 females and 6 males, living in the Gothenburg area. These healthy control subjects were randomly recruited among friends to the participating individuals with T1D and relatives to the hospital staff. The current study commenced in April 2019, and was completed by October 2019. Clinical data of subjects with T1D and healthy controls are presented in Table 1. Exclusion criteria were obesity, celiac disease, hypothyroidism, metabolic, skeletal and inflammatory diseases, breastfeeding and pregnancy.

**Table 1:** Clinical data of subjects with T1D and matched healthy controls.

	T1D (n = 12)	Controls (n = 12)	*P*-value
Age (years)	16.4 (0.9) 16.5 (15.0; 17.9)	16.6 (0.8) 16.6 (15.0; 17.5)	0.63
Sex (females / males)	5 / 7	6 / 6	
Weight (kg)	68.3 (10.4) 68.5 (50.6; 87.0)	66.9 (7.3) 67.5 (56.3; 80.4)	0.70
Height (m)	1.73 (0.11) 1.72 (1.56; 1.90)	1.76 (0.05) 1.73 (1.70; 1.85)	0.51
BMI (kg/m^2^)	22.6 (1.8) 22.0 (19.8; 26.0)	21.7 (2.3) 21.5 (18.8; 26.4)	0.27
Comorbidities (other than T1D)	Allergy for pollen, mite (n = 3) Asthma (n = 1) Psoriasis (n = 1) ADHD (n = 1)	Allergy for pollen, mite (n = 1) Eczema (n = 1)	
Medications (other than insulin)	Asthma/allergy (n = 3) Dexamphetamine (n = 1)	Allergy (n = 1) Local steroid (n = 1) Oral contraceptives (n = 1)	

For categorical variables n (%) is presented. For continuous variables, mean (SD) and median (minimum; maximum) are presented. *P*-values calculated by Student’s t-test.

*BMI* body mass index.

Sample size for miRNA analysis was derived from power analysis, which was performed using G*Power Version 3.1.9.6. based on a Wilcoxon-Mann–Whitney test (two groups) model. We assumed equal group sizes (N2/N1 = 1), α error probability of 0.05, a power (1-β error probability) of 0.80, and an effect size of 1.33, which resulted in a suggested total sample size of n = 22 (11 per group) achieving an actual power of 0.826.

The present study was approved by the regional research ethics committee of the University of Gothenburg (No. 1076-18) and conducted in accordance with the 1964 Helsinki declaration and its later amendments. All adolescents and their parents received oral and written information prior to study entry, and written consent was obtained. Both study and control subjects were enrolled at The Queen Silvia Children’s Hospital in Gothenburg, Sweden, and all clinical investigations and blood sampling were performed during one visit.

### Assessment of body composition

Body composition was assessed by dual-energy X-ray absorptiometry (DXA) Lunar iDXA (GE Lunar Corp., Madison, WI, USA). Measurements by peripheral quantitative computed tomography (pQCT) were performed on the left tibia at 4% and 66% of the tibia length using the XCT 2000 (Stratec Medizintechnik GmbH, Pforzheim, Germany) with software version 6.00 as reported elsewhere^11^.

### miRNA analysis

EDTA plasma samples were collected and directly placed on wet ice. Samples were centrifuged 1000×*g* at 4 °C for 10 min within 30 min of sampling. The supernatant was transferred to a new tube and centrifuged at 3800×*g* at 4 °C for 15 min. Aliquots of EDTA plasma were transferred, within 30 min of centrifugation, into tubes that were immediately stored at − 80 °C until analyses.

Total RNA extraction from 200 µL plasma samples was performed using the miRNeasy Mini Kit (Qiagen, Germany)^12^. Plasma was thawed on ice and centrifuged at 12,000×*g* for 5 min to remove any cellular debris. For sample lysis, 200 µL of plasma were mixed with 1000 µL Qiazol to which 1 µL spike-in controls had been added, which contains a mix of three spike-ins termed UniSp2, 4, and 5 that cover a 10,000 fold range (~ 13 LOG2, i.e., Cq-values) (Exiqon, Denmark). Following incubation at room temperature for 10 min, 200 µL chloroform were added to the lysates. After centrifugation at 12,000×*g* for 15 min at 4 °C, 650 µL of the upper aqueous phase were obtained and mixed with 7 µL glycogen (50 mg/mL) to enhance precipitation. The aqueous phase was then transferred to a miRNeasy mini column, and RNA was precipitated by adding 750 µL ethanol. Washing with RPE and RWT buffer was performed in a QiaCube liquid handling robot (Qiagen, Germany). In the last step, total RNA was eluted in 30 µL nuclease free water and stored at − 80 °C until further analysis.

RT-qPCR analysis of 187 distinct miRNAs and 5 controls was performed as previously described^12–15^. In brief, cDNA was synthesized using the Universal cDNA Synthesis Kit II using reaction conditions provided by the manufacturer (Exiqon, Denmark). In total, 2 µL of total RNA were used as input per 10 µL reverse transcription (RT) reaction mix. Cel-miR-39-3p, which is part of the Universal cDNA Synthesis Kit II, was added to each RT reaction to monitor RT efficiency. PCR amplification was performed in a 384-well plate format using custom Pick&Mix plates (Exiqon, Denmark) in a Roche LC480 II instrument (Roche, Germany) and EXiLENT SYBR Green mastermix (Exiqon, Denmark) with the following settings: 95 °C for 10 min, 45 cycles of 95 °C for 10 s and 60 °C for 60 s, followed by melting curve analysis. Cycle of quantification values (Cq-values) were determined using the second derivative method as provided by the Roche LC480 software. Data quality was assessed by visual inspection of spike-in control data. Hemolysis was assessed in all samples using the ratio of miR-23a-3p versus miR-451a according to Blondal et al.^16^ and applying a cut-off of > 7 to the ratio for calling a sample hemolytic.

A description of the qPCR plate design, methodology, as well as all raw and normalized data have been deposited at NCBI Gene Expression Omnibus and can be accessed under the number GSE226755.

### Statistical analysis

Differences in selected clinical parameters between the case and control groups were assessed with Student’s t-test (two sided; unpaired) after confirming normal distribution of the variables. RT-qPCR miRNA data was normalized to UniSp4 spike in Cq-values after checking the comparability of spike-in normalization to global mean (GM) normalization and normalization using a previously reported reference miRNA (miR-320d)^17^. Clinical data were complete. Missing miRNA data below the detection limit were not imputed. For visualization and unsupervised clustering of miRNA levels in the samples, a heatmap of univariate scaled and centered Cq-values were plotted using ClustVis v2.10.0^18^ (https://biit.cs.ut.ee/clustvis/). A heatmap representing a hierarchical cluster analysis conducted upon a Spearman correlation network of miRNA levels was generated using the R package ComplexHeatmaps (https://bioconductor.org/packages/release/bioc/html/ComplexHeatmap.html)^19^. Correlations between miRNA expression and continuous variables were investigated using Spearman correlations. For categorical variables, miRNA effects were tested using Mann–Whitney U tests. In addition, combinations of categorical variables were analyzed by an initial Kruskal–Wallis test and in case of significant results, followed by Mann–Whitney U tests of all combinations. For all statistical analysis, *P*-values were calculated and subsequently adjusted for multiple testing with the false discovery rate (FDR) method.

Tissue specificity indices (TSI) were obtained from the Human miRNA Tissue Atlas^20^. Mean and standard deviation were calculated from TSI-values for raw, variance stabilized normalization, and quantile normalized microarray data.

Messenger RNA targets of selected miRNAs were analyzed using miRNet v2.0^21^. The list of miRNA miRBase^22^ IDs was uploaded through the web-interface and analyzed against experimentally verified human targets deposited in miRTarbase v8.0^23^ and the resulting gene target network was analyzed for overrepresentation (“enrichment”) using a hypergeometric distribution test and Kyoto Encyclopedia of Genes and Genomes (KEGG) pathway annotations^24^.

## Results

### Registry data and body composition

Clinical data and biochemical assessments from the SWEDIABKIDS registry are presented in Table 2. The mean (SD) age at T1D diagnosis was 5.3 (2.0) years with a mean diabetes duration of 11.1 (2.0) years.

**Table 2:** Clinical data of subjects with T1D.

	T1D (n = 12)	
Age at diabetes onset (years)	5.3 (2.0) 5.6 (2.9; 8.7)	
Diabetes duration (years)	11.1 (2.0) 10.8 (8.0; 15.0)	
Continuous glucose monitoring (n)	12	
Continuous subcutaneous insulin infusion pump (n)	9	

HbA1c according to age	IFCC (mmol/mol)	NGSP (%)
Last HbA1c measurement	53.3 (8.3) 52.0 (39.0; 67.0)	7.0 (2.9) 6.9 (5.7; 8.3)
HbA1c, 0–8.9 years	56.2 (7.2) 55.8 (44.7; 69.0)	7.3 (2.8) 7.3 (6.2; 8.5)
HbA1c, 9.0–13.9 years	56.6 (9.1) 55.6 (42.4; 77.7)	7.3 (3.0) 7.2 (6.0; 9.3)
HbA1c, 14.0–17.9 years	55.5 (9.7) 53.7 (38.1; 74.5)	7.2 (3.0) 7.1 (5.6; 9.0)
HbA1c, 0–17.9 years	56.1 (7.8) 56.1 (44.0; 73.4)	7.3 (2.9) 7.3 (6.2; 8.9)

Continuous glucose monitoring, real time or intermittent scanning. For continuous variables, mean (SD)/median (minimum; maximum) are presented.

*HbA1c* glycated hemoglobin A1c, *IFCC* International Federation of Clinical Chemistry, *NGSP* National Glycohemoglobin Standardization Program.

The mean glycated hemoglobin A1c (HbA1c) value during different age periods are presented in Table 2. The study participants were generally well controlled during the entire age span from the diabetes diagnosis: mean (SD) HbA1c of the last measurement before the study was 53.3 mmol/mol (8.3), 7.0% (2.9). All 12 study participants used continuous glucose monitoring and 9 of 12 used continuous subcutaneous insulin infusion pumps.

No significant differences were found between the study and control groups for body composition parameters assessed by DXA and pQCT (Table 3).

**Table 3:** DXA and pQCT of subjects with T1D and matched healthy controls.

	T1D (n = 12)	Controls (n = 12)	*P*-value
DXA measurements			
Left leg fat mass (%)	30.3 (7.9) 30.9 (15.7; 40.5)	27.7 (7.2) 27.2 (16.2; 41.0)	0.41
Trunk fat mass (%)	24.7 (7.3) 23.9 (13.9; 38.0)	21.2 (8.9) 21.3 (9.0; 37.7)	0.30
Total body (less head), fat mass (%)	27.5 (7.7) 27.5 (14.8; 39.3)	24.2 (8.0) 24.2 (13.3; 39.3)	0.32
Total body (less head), lean mass (g)	44,650 (9,125) 47,151 (32,036; 57,036)	45,526 (6,435) 46,472 (36,479; 55,863)	0.79
pQCT measurement			
Fat/muscle area ratio (%)	30.92 (21.93) 37.95 (0.00; 56.00)	37.70 (12.33) 36.64 (20.01; 63.27)	0.38

For continuous variables, mean (SD)/median (minimum; maximum) are presented.

*P*-values calculated by Student’s t-test.

*DXA* dual-energy X-ray absorptiometry, *pQCT* peripheral quantitative computed tomography.

### Circulating miRNA regulation in children with long duration T1D versus controls

RT-qPCR data for circulating miRNAs can be biased by pre-analytical and analytical variability originating from low sample quality (hemolysis), presence of inhibitors, or assay variability. To assess data quality, we visualized results obtained for RNA, RT, and PCR spike-in controls (Suppl. Fig. S1A,B) and observed low variability and no significant outliers. Next, we calculated the hemolysis ratio of miR-23a/miR-451a and plotted the results (Suppl. Fig. S1C). We observed homogeneous distribution between 5 and 7 and no extreme values. Therefore, no samples were filtered from the data set due to low data quality.

**Figure 1:**
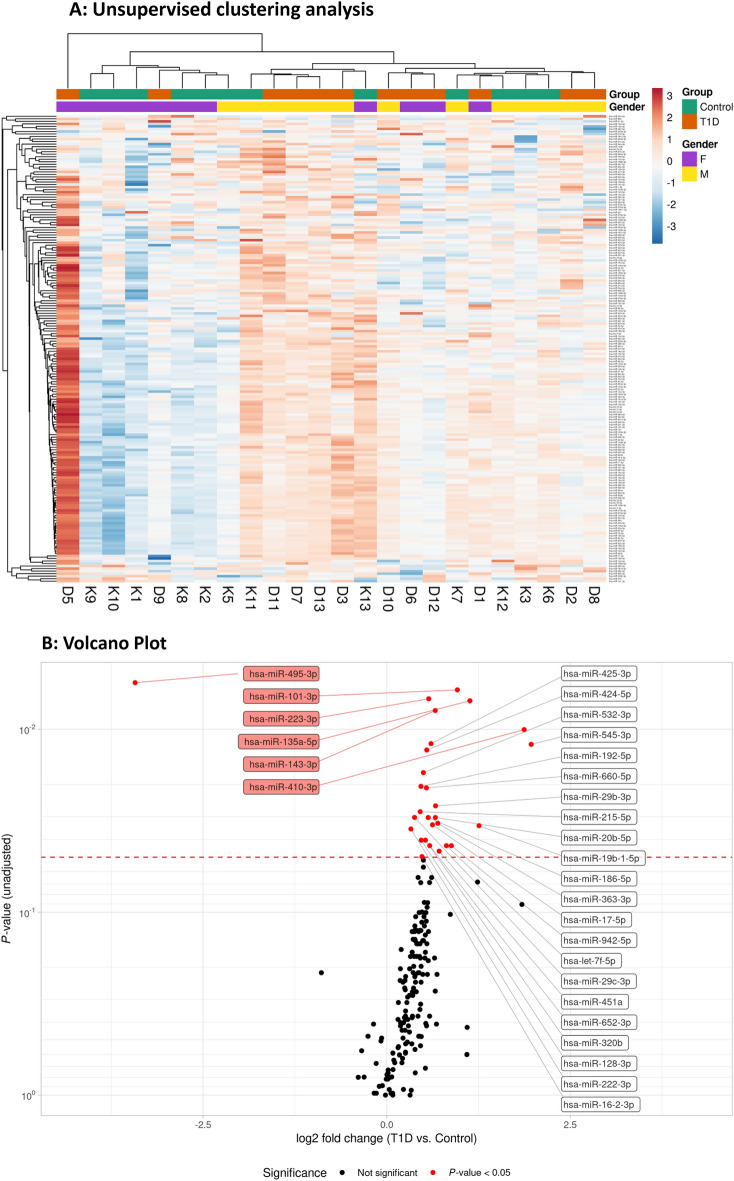
(**A**) Hierarchical clustering and representation as heatmaps. Heatmap illustrating expression levels of 187 miRNAs levels in 24 samples (12 T1D (D) and 12 controls (K)). Gender and group information is provided for each sample. Pearson correlation and average linkage was used for rows (miRNAs). Euclidean distance and complete linkage were used for columns (samples). This heatmap was generated using ClustVis v2.10.0^18^ (https://biit.cs.ut.ee/clustvis/). *F* female, *M* male, *T1D* type 1 diabetes. (**B**) The volcano plot depicts the log2-transformed fold change in circulating miRNA levels in T1D subjects in comparison with controls (x-axis) in relation to the unadjusted *P*-value (y-axis). The higher up and further left/right a miRNA is plotted, the larger difference in expression between T1D and controls. We observed that 27 (out of 187) miRNAs were upregulated and 1 was downregulated in plasma from individuals with T1D in comparison with the control group. The 6 miRNAs with *P*-values of ≤ 0.01 are highlighted (red boxes). The dashed line represents the *P*-value of 0.05.

Unsupervised data analysis (hierarchical clustering) based on 187 detected miRNAs did not indicate a clear grouping of samples according to sex or T1D (group) (Fig. 1A). One individual with diabetes (D5) exhibited the highest levels in the heatmap analysis for the majority of miRNAs, which differed from the other children with diabetes (Fig. 1A). The quality control data (Suppl. Fig. S1) did not suggest technical issues that could explain the observed differences in expression levels, and available clinical and phenotypic data did not indicate any noteworthy abnormalities. Hence, sample D5 was retained in the analysis.

**Figure 2:**
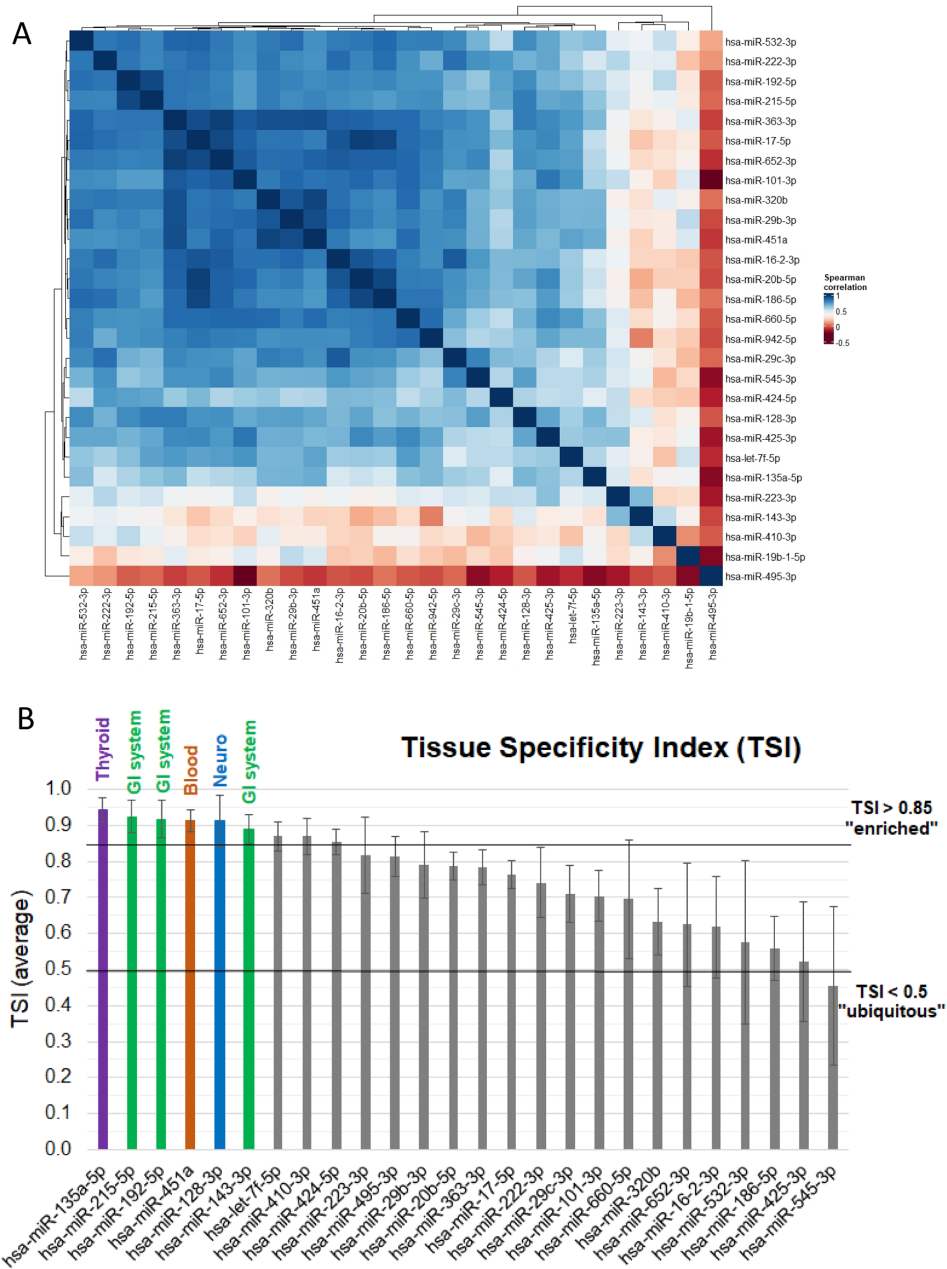
Clusters and correlation of differentially expressed circulating miRNAs and TSI. (**A**) The heatmap represent a hierarchical cluster analysis conducted upon a Spearman correlation network of miRNA levels that were found differentially regulated in T1D compared to control subjects (n = 28). This heatmap was generated using the R package ComplexHeatmaps (https://bioconductor.org/packages/release/bioc/html/ComplexHeatmap.html)^19^. (**B**) TSI were obtained from the Human miRNA Tissue Atlas (https://ccb-web.cs.uni-saarland.de/tissueatlas/) for all differentially regulated miRNAs except miR-942-5p, for which no TSI data was available (n = 27). MiRNAs with tissue enrichment (TSI > 0.85) were highlighted in red (blood), purple (thyroid), green (gastrointestinal (GI) system), and blue (neuro).

By performing differential expression analysis (*P* < 0.05), we observed that 27 (out of 187) miRNAs were upregulated and 1 miRNA was downregulated in plasma from individuals with T1D in comparison with the control group (Fig. 1B). The adjusted p-value (FDR) in this list reached 0.30, which means that we identified 28 candidate miRNAs with a risk of 30% (8 out of 28) false discoveries. No adjustments were made in the analysis for age, sex and BMI since the groups were well matched (Table 1). The 28 candidate miRNAs were found to include several groups of positively correlated miRNAs with significantly elevated plasma levels in T1D (Fig. 2A). We used the TSI-values provided by the Human miRNA Tissue Atlas^20^ (https://ccb-web.cs.uni-saarland.de/tissueatlas/ ) to assess whether any of the differentially regulated miRNAs were known to be tissue-enriched (TSI-value > 0.85). Indeed, we identified six tissue-enriched miRNAs in our list, of which three (hsa-miR-143-3p, hsa-miR-192-5p, hsa-miR-215-5p) are enriched in the gastrointestinal system, one (hsa-miR-135a-5p) in thyroid tissue, one (hsa-miR-451a) in blood cells, and one (hsa-miR-128-3p) in nervous tissue (Fig. 2B, Suppl. Fig. S2). Finally, we compared the log_2_ fold changes (LFC) between T1D and controls obtained after spike-in normalization to LFCs obtained after GM, miR-320d, and no normalization. We observed high correlation of LFCs, however, a shift in the LFCs to < 0 for GM normalization, which was not found for spike-in, miR-320d, or no normalization (Suppl. Fig. S3A).

**Figure 3:**
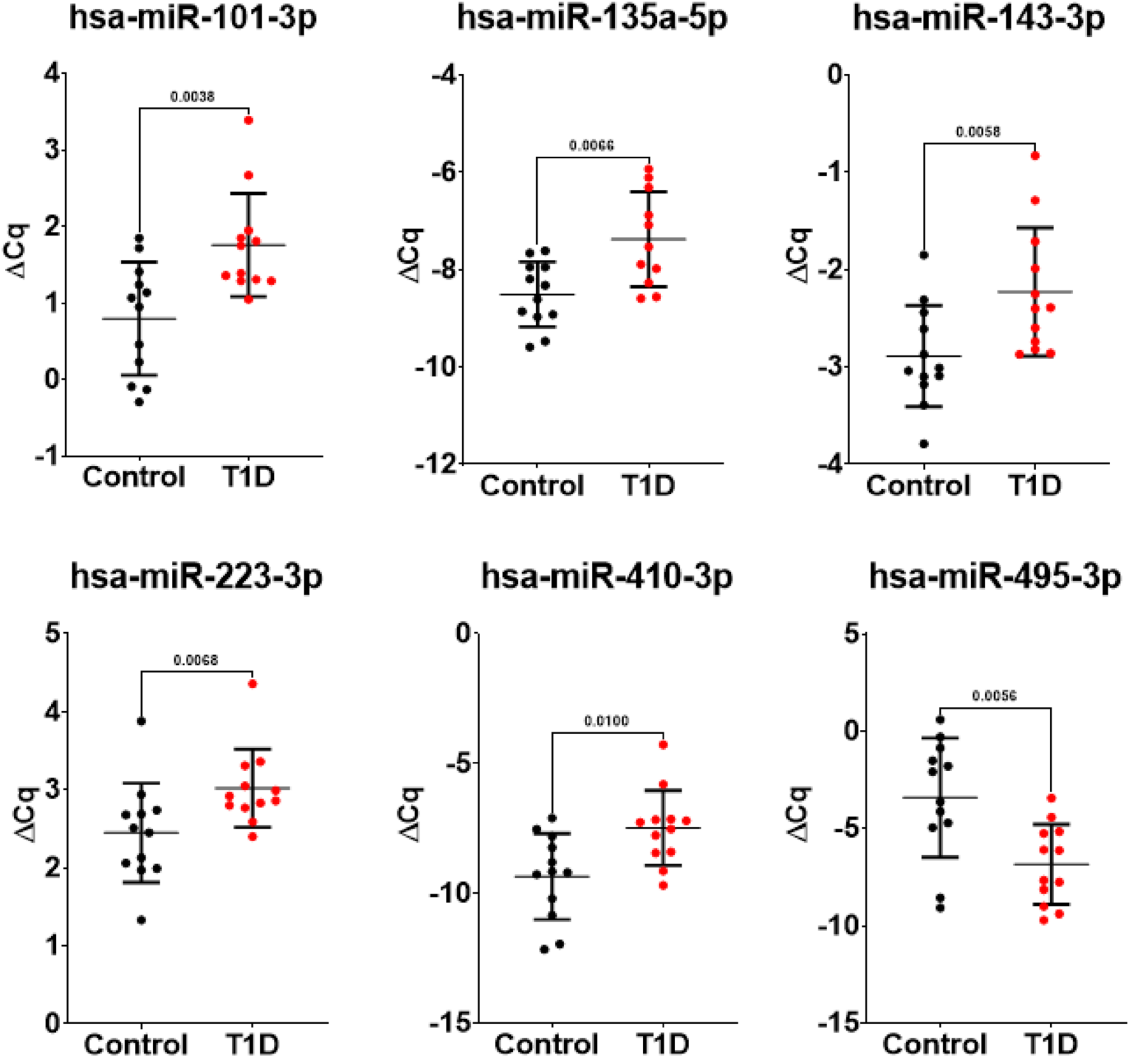
Scatter plots representing the spike-in normalized (ΔCq) for the 6 miRNAs with *P*-values of ≤ 0.01.

Six miRNAs with *P*-values below 0.01 and robust difference independent of the normalization approach (Suppl. Fig. S3B), five up-regulated (hsa-miR-101-3p, hsa-miR-135a-5p, hsa-miR-143-3p, hsa-miR-223-3p and hsa-miR-410-3p) and one down-regulated (hsa-miR-495-3p), were selected for further analysis (Fig. 3). Previously reported data and suggested functional roles for the 6 selected miRNAs with *P*-values of ≤ 0.01 are presented in Table 4.

**Table 4:** Previously reported data and role for the six selected miRNAs with *P*-values of ≤ 0.01.

miRNA-ID	Regulation in T1D subjects (down or up)	Role and proposed function Literature search: word cloud from miRBase^22^	miRBase references (accessed on May 5, 2023)
hsa-miR-101-3p	Up-regulated		307 publications
hsa-miR-135a-5p	Up-regulated		153 publications
hsa-miR-143-3p	Up-regulated		482 publications
hsa-miR-223-3p	Up-regulated		400 publications
hsa-miR-410-3p	Up-regulated		59 publications
hsa-miR-495-3p	Down-regulated		50 publications

*miRNA* microRNA.

We found no significant correlations between the 28 candidate miRNAs (observed in the differential expression analysis, *P* < 0.05, FDR < 0.30), and continuous variables in the study and control groups: age, weight, height, BMI, left leg fat mass, trunk fat mass, total body (less head) fat mass, total body (less head) lean mass and fat/muscle area ratio. No significant correlations (FDR < 0.05) were found for the study group between the 28 miRNAs and the reported continuous variables: diabetes duration, HbA1c last visit, insulin dosage last visit, glucose average last visit, glucose SD last visit, average HbA1c last year, average HbA1c 14–17.9 years and average HbA1c 0–17.9 years.

**Figure 4:**
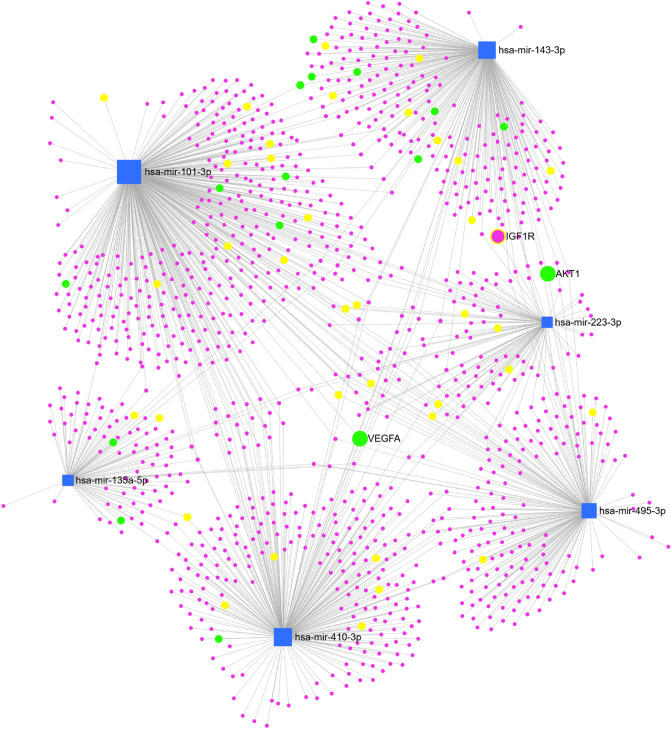
Network diagram displaying the interactions between six miRNAs showing dysregulation in plasma of children with T1D (*P* < 0.01) and their messenger RNA targets. Blue boxes are miRNAs, purple circles are messenger RNAs. Yellow-labeled messenger RNAs are associated with the MAPK signaling pathway. Green labeled messenger RNAs are associated with VEGF signaling pathway.

Finally, we analyzed known messenger RNA target genes of our six miRNA candidates using miRNet 2.0. We identified 1127 human messenger RNA targets with experimental evidence for interactions with the six selected miRNA candidates (Fig. 4). Of these, 112 genes were found to share interactions with at least two of the six selected miRNAs, and eight genes were regulated by three miRNAs (Suppl. Table 1). KEGG pathway enrichment analysis (Suppl. Table 2) identified vascular endothelial growth factor (VEGF) signaling pathway (16 genes, FDR < 0.001) and mitogen-activated protein kinase (MAPK) signaling pathway (43 genes, FDR < 0.0001) genes to be enriched in the list of experimentally verified targets, including VEGFA (targeted by hsa-miR-101-3p, hsa-miR-135a-5p, and hsa-miR-410-3p), IGF-1 receptor (targeted by hsa-miR-143-3p and hsa-miR-223-3p), and AKT serine/threonine kinase 1 (AKT1) (targeted by hsa-miR-143-3p and hsa-miR-495-3p).

### Circulating miRNA levels by sex

We compared the circulating miRNAs between males and females. Six out of 28 miRNAs differed between cases and controls (hsa-miR-16–2-3p, hsa-miR-29b-3p, hsa-miR-29c-3p, hsa-miR-222-3p, hsa-miR-320b, hsa-miR-532-3p), showed significant (*P* < 0.05, FDR < 0.20, Kruskal–Wallis test) sex differences (Suppl. Fig. S4). None of these showed significant tissue enrichment, TSI > 0.85 (Fig. 2B). A potential interaction between sex and diabetes could not be tested due to the small sample size.

## Discussion

This is the first study, to our knowledge, exploring whether expression of miRNA differs between adolescents with long-term T1D (mean duration 11.1 years) and healthy controls. Twenty-seven (out of 187) endogenous miRNAs were upregulated and one miRNA was downregulated in plasma from individuals with T1D in comparison with healthy controls. The majority of the identified miRNAs (n = 28) were found to be ubiquitously expressed across tissues according to the Human miRNA Tissue Atlas (Fig. 2B), with the exception of six miRNAs that we found to be enriched in the gastrointestinal system, thyroid tissue, blood cells and nervous tissue. Further analysis found six miRNAs associated with long duration T1D (*P*-values below 0.01) where five were up-regulated and one was down-regulated (Fig. 3).

A recent meta-analysis identified seven studies investigating miRNA in T1D in children and adolescents soon after diagnosis (time span of two days to one year)^25^. Two miRNAs were upregulated in T1D, i.e., miR-181 and 210, and one miRNA, miR-375, was upregulated in control individuals. We could not confirm these findings, which could be due to different metabolic effects in long-term diabetes duration in comparison with the more acute effects soon after diagnosis.

To further explore the genetic pathway, we identified 1127 human messenger RNA targets with experimental evidence for interactions with the six selected miRNA candidates (Fig. 4). The KEGG pathway enrichment analysis identified these six miRNAs to be associated with targets such as VEGFA, IGF-1 receptor and AKT1. VEGF has been identified as a primary initiator of proliferative diabetic retinopathy, but is also associated with the development of neuropathy and nephropathy in diabetes^26^. The IGF-1 receptor plays a role in cell growth and glucose regulation, and can potentially induce pathological disorders such as cardiovascular disease and cancer^27,28^. AKT1 is important in several metabolic actions of insulin and regulation of β-cell mass^29^.

Overexpression of hsa-miR-101-3p has been demonstrated in adolescents with recent-onset T1D^30^. The meta-analysis by Margaritis et al.^25^ analyzed hsa-miR-101-3p data from two studies^10,30^ and found a trend between healthy controls and T1D. Even though our study comprises individuals with long-duration T1D, the overexpression of hsa-miR-101-3p seems to be consistent in all subjects. Previously reported data from the miRBase (Table 4) indicates an association between hsa-miR-101-3p and the enzyme enhancer of zeste homolog 2 (EZH2), which is involved in methylation of histones. EZH2 inhibits genes involved in tumor suppression, and overexpression or mutation of the *EZH2* gene has been linked to cancer^31^.

Both experimental and clinical studies have identified hsa-miR-135a as a treatment target for renal fibrosis in diabetic nephropathy^32,33^. The observed upregulation of hsa-miR-135a suggests a mechanism that could, directly or indirectly, influence the development of renal fibrosis in individuals with diabetes. It has also been shown that hsa-miR-135a, among other miRNAs, is of importance for reprogramming acinar cells into insulin producing cells^34^, which suggests a therapeutic potential for this miRNA. Hsa-miR-135a is a pivotal miRNA in biogenesis and regulation in various forms of cancer^35^. Several signaling pathways, e.g., the MAPK and JAK2/STAT3 pathways are involved in hsa-miR-135a-mediated cell proliferation and cancer progression. The rationale for targeting hsa-miR-135a in cancer-related therapy is evident, which could improve the outcome for individuals with cancer or at risk for developing cancer.

Hsa-miR-143 is one of the top nine reported miRNAs associated in atherosclerotic disease and hypertension^36^, and it has been reported that hsa-miR-143 modulate the function of vascular smooth muscle cells and thereby contributing to the development and progression of arteriosclerosis^37^. The upregulated levels of hsa-miR-143 in the current study could indicate an early effect on vascular smooth muscle cells already in adolescent individuals with T1D. Lan et al.^38^ described a specific modulation of hsa-miR-143 in the regulation of specific targets such as IRS-1, ORP8 and the IGF-1 receptor in the insulin signaling pathway, which was confirmed for the IGF-1 receptor in the network diagram (Fig. 4). An important role for hsa-miR-143 has also been demonstrated in glucose uptake and insulin signaling in vascular smooth muscle cells^38^. The miRBase (Table 4) shows the highest association between hsa-miR-143 and hsa-miR-145, which is a miRNA that also targets insulin signaling and is associated with atherosclerotic disease^36–38^. Both hsa-miR-143 and hsa-miR-145 have a profound role in tumorigenesis and progression of various neoplasms, and their therapeutic potential as treatment targets has been proposed^39^.

Circulating hsa-miR-223 has been associated with type 1 and 2 diabetes, obesity, inflammation, autoimmunity, diabetic nephropathy and retinopathy^40,41^. Hsa-miR-223-3p is abundant in platelets, released during platelet activation and has been suggested as a biomarker in cardiovascular disease^42^. The review by Gangwar et al.^36^ showed that hsa-miR-223 is one of the most significant miRNAs associated with atherosclerotic disease and hypertension. In contrast to the present study, Garavelli et al.^43^ did not find any difference between persons with T1D and healthy controls for hsa-miR-223.

This is the first study to demonstrate a difference for hsa-miR-410-3p between individuals with T1D and control subjects. One of the few clinical studies on hsa-miR-410 showed significantly higher expression in prostate cancer patients in comparison with healthy controls^44^. The review by Wen et al.^45^ on hsa-miR-410 showed evidence of regulation of genes that both can promote and suppress cancer. Hsa-miR-410 negatively regulates The Solute Carrier Family‐34, Member‐2 (a.k.a. SLC34A2), a Na^+^‐dependent phosphate transporter protein, which plays a pivotal role in carcinogenesis^45^. Previously reported data from the miRBase (Table 4) shows also an association with SLC34A2.

Hsa-miR-495-3p was the only down-regulated miRNA in this study. Previous studies have shown that this miRNA is associated with various developmental, inflammatory, immunological processes^46^ in healthy tissue, and it is also involved in proliferation and metastasis of cancer cells^47^. Hsa-miR-495-3p was one of 18 (out 723) urinary miRNAs that was associated with the subsequent development of microalbuminuria in T1D^48^. Furthermore, hsa-miR-495-3p was investigated in human cardiac fibroblasts, and it was demonstrated that overexpression of miR-495 has a protective effect in cases of high glucose-induced cardiac fibrosis^49^. Although rather few studies about hsa-miR-495-3p, the miRBase (Table 4) shows the highest association with *glucose*, which warrants further exploratory studies on this particular miRNA in T1D.

It is noteworthy that we found several associations with cancer for the six miRNAs with the largest difference in plasma, although the association between diabetes and cancer has been suggested for over a century^50^. A recent meta-analysis showed that there was a higher incidence of cancer among individuals with diabetes in comparison with a cancer-free population (RR 1.42, CI 1.30–1.54)^51^. An epidemiological analysis identified 313,907 matched individuals with and without diabetes between 2001 to 2018 in England^52^. This analysis showed that there has been a decline in vascular complications and shift to cancer as the leading cause of diabetes-related death. Although an extensive epidemiological analysis, it should be noted that it was not possible to correctly distinguish type 1 from type 2 diabetes consistently over the 18-year period. In addition, the number of individuals with T1D would have been underpowered to investigate findings relating to cause-specific mortality. The linkage to cancer and cancer-related mortality, specifically for T1D, has also been shown in other studies with sufficiently powered study populations^53,54^, which supports the objective to further investigate miRNAs as biomarkers and potential treatment targets. The current study demonstrates associations between cancer and all of the six miRNAs significantly associated with long duration T1D (*P*-values below 0.01).

The strength of this study is the long diabetes duration with a mean of 11.1 years in a group of adolescents in a narrow age span with registered metabolic data.** S**trict exclusion criteria were applied at the enrollment, and the groups were well-matched regarding age, sex and body composition. A state-of-the art RT-qPCR protocol^12,13^ was used for analysis of 187 distinct circulating miRNAs. Known pre-analytical and analytical sources of variability such as hemolysis, RT-qPCR inhibition, and lack of homogenous RNA recovery^7^ were successfully controlled, resulting in a high-quality data set with low analytical variability that is suitable for assessing miRNA variability in the context of T1D.

Although a homogenous group of individuals, a larger study group would have been preferable to increase the statistical power. A possible bias could come from the recruitment of persons with T1D, where only the most motivated individuals participated (possibly well-controlled cases) due to the additional investigations during the clinical follow-up visit. To overcome this possible bias, an extra effort was made to invite all individuals at the clinical site who fulfilled the inclusion criteria.

In conclusion, six miRNAs differed between young subjects with long-duration T1D and healthy controls; five were up-regulated (hsa-miR-101-3p, hsa-miR-135a-5p, hsa-miR-143-3p, hsa-miR-223-3p and hsa-miR-410-3p) and one down-regulated (hsa-miR-495-3p). This is the first study that demonstrates a difference for hsa-miR-410-3p. We found several associations with cancer for the six miRNAs with the largest difference between persons with T1D and controls, which warrants further investigation as biomarkers and potential treatment targets. TSI-values, provided by the Human miRNA Tissue Atlas, showed that changes in miRNAs occur across several tissues, specifically in blood cells, gastrointestinal, nerve, and thyroid tissues. Functional analysis identified AKT1, VEGFA, and IGF-1 receptor as targets for important biological pathways including angiogenesis, cell proliferation and growth signaling. This study contributes with new knowledge about miRNAs as novel biomarkers that could serve in the clinical setting in individuals with T1D. Circulating miRNAs are potential treatment targets with clinical implications comprising personalized approaches to reduce diabetes-related long-term complications.

## Supplementary Information

Supplementary Tables.

Supplementary Figure 1.

Supplementary Figure 2.

Supplementary Figure 3.

Supplementary Figure 4.

## Data Availability

The datasets generated and/or analyzed during the current study are available in the NCBI Gene Expression Omnibus (GEO) data repository and can be accessed under the record GSE226755.

## Abbreviations

AKT1: AKT serine/threonine kinase 1
BMI: Body mass index
Cq-values: Cycle of quantification values
DXA: Dual-energy X-ray absorptiometry
FDR: False discovery rate
GM: Global mean
HbA1c: Glycated hemoglobin A1c
IFCC: International Federation of Clinical Chemistry
IGF: Insulin-like growth factor
KEGG: Kyoto Encyclopedia of Genes and Genomes
LFC: Log_2_ fold change
MAPK: Mitogen-activated protein kinase
miRNA: MicroRNA
NGSP: National Glycohemoglobin Standardization Program
pQCT: Peripheral quantitative computed tomography
RT: Reverse transcription
RT-qPCR: Reverse transcription quantitative PCR
SWEDIABKIDS: Swedish Pediatric Diabetes Quality Registry
T1D: Type 1 diabetes
TSI: Tissue specificity index
VEGF: Vascular endothelial growth factor
