# Supplementary material for: Circulating microRNAs in young individuals with long-duration type 1 diabetes in comparison with healthy controls
Source: Sci Rep. 2023 Jul 19;13:11634. doi: 10.1038/s41598-023-38615-7 (PMC10356803; doi:10.1038/s41598-023-38615-7)
Supplement: Supplementary file 1 — Supplementary Tables. [file 41598_2023_38615_MOESM1_ESM.docx]

**Supplementary Table 1.** List of eight experimentally confirmed messenger RNA targets with at least three of the top six dysregulated miRNAs (*P* < 0.01) in type 1 diabetes.

| **Gene Symbol** | **hsa-miR-101-3p** | **hsa-miR-135a-5p** | **hsa-miR-143-3p** | **hsa-miR-223-3p** | **hsa-miR-410-3p** | **hsa-miR-495-3p** |
| --- | --- | --- | --- | --- | --- | --- |
| MDM2 |  |  | X | X | X |  |
| NUFIP2 | X | X |  |  | X |  |
| PPP2R5E | X |  | X |  | X |  |
| RREB1 | X |  | X |  | X |  |
| SLC39A6 | X | X |  |  | X |  |
| TNPO1 | X |  |  |  | X | X |
| TTYH3 |  |  | X |  | X | X |
| VEGFA | X |  |  |  | X | X |

**Supplementary Table 2.** List of 67 KEGG pathways (Kanehisa et al., Protein Sci. 2022;31:47-53. <https://doi.org/10.1002/pro.4172> ) in which the 1127 genes targeted by the top six miRNAs (*P* < 0.01) that were found to be overrepresented.

| **KEGG Pathway** | **Identified Gene Targets** | ***P*-value** | **Adjusted**  ***P*-value** |
| --- | --- | --- | --- |
| Pathways in cancer | 69 | 8.05E-19 | 8.05E-17 |
| Colorectal cancer | 23 | 2.63E-14 | 1.32E-12 |
| Prostate cancer | 30 | 6.86E-14 | 2.29E-12 |
| Focal adhesion | 45 | 1.36E-12 | 3.40E-11 |
| Chronic myeloid leukemia | 25 | 1.10E-11 | 2.20E-10 |
| Glioma | 23 | 3.51E-11 | 5.85E-10 |
| Melanoma | 23 | 1.01E-10 | 1.44E-09 |
| Pancreatic cancer | 23 | 1.42E-10 | 1.78E-09 |
| Endometrial cancer | 18 | 3.18E-10 | 3.53E-09 |
| Neurotrophin signaling pathway | 30 | 1.26E-09 | 1.26E-08 |
| Bladder cancer | 14 | 2.23E-09 | 2.03E-08 |
| ErbB signaling pathway | 24 | 4.39E-09 | 3.66E-08 |
| Acute myeloid leukemia | 19 | 6.02E-09 | 4.63E-08 |
| HTLV-I infection | 37 | 4.28E-08 | 3.06E-07 |
| Jak-STAT signaling pathway | 24 | 6.95E-08 | 4.63E-07 |
| Renal cell carcinoma | 18 | 9.78E-08 | 6.11E-07 |
| Small cell lung cancer | 21 | 1.08E-07 | 6.35E-07 |
| MAPK signaling pathway | 43 | 2.00E-07 | 1.11111E-06 |
| Non-small cell lung cancer | 16 | 3.48E-07 | 1.75238E-06 |
| Chemokine signaling pathway | 34 | 3.60E-07 | 1.75238E-06 |
| Hepatitis C | 23 | 3.68E-07 | 1.75238E-06 |
| Toxoplasmosis | 22 | 3.95E-07 | 1.79546E-06 |
| Regulation of actin cytoskeleton | 33 | 4.48E-07 | 1.94783E-06 |
| Epstein-Barr virus infection | 20 | 0.00000461 | 1.92083E-05 |
| Osteoclast differentiation | 23 | 0.000009 | 0.000036 |
| mTOR signaling pathway | 13 | 0.00000981 | 3.77308E-05 |
| Insulin signaling pathway | 25 | 0.0000106 | 3.92593E-05 |
| Toll-like receptor signaling pathway | 20 | 0.0000129 | 4.60714E-05 |
| T-cell receptor signaling pathway | 20 | 0.0000151 | 5.2069E-05 |
| p53 signaling pathway | 16 | 0.000017 | 5.66667E-05 |
| Adherens junction | 16 | 0.0000251 | 8.09677E-05 |
| Progesterone-mediated oocyte maturation | 17 | 0.0000385 | 0.000120313 |
| Chagas disease (American trypanosomiasis) | 18 | 0.0000461 | 0.000139697 |
| B-cell receptor signaling pathway | 16 | 0.000062 | 0.000182353 |
| VEGF signaling pathway | 16 | 0.0000735 | 0.000206389 |
| TGF-beta signaling pathway | 17 | 0.0000743 | 0.000206389 |
| Dorso-ventral axis formation | 6 | 0.000085 | 0.00022973 |
| Measles | 19 | 0.0000931 | 0.000245 |
| Bacterial invasion of epithelial cells | 13 | 0.000123 | 0.000315385 |
| Fc gamma R-mediated phagocytosis | 18 | 0.00015 | 0.000375 |
| Phosphatidylinositol signaling system | 15 | 0.000226 | 0.00055122 |
| Type 2 diabetes mellitus | 11 | 0.00046 | 0.001086047 |
| Cell cycle | 20 | 0.000467 | 0.001086047 |
| NOD-like receptor signaling pathway | 11 | 0.000556 | 0.001263636 |
| Thyroid cancer | 8 | 0.000576 | 0.00128 |
| Fc epsilon RI signaling pathway | 14 | 0.000761 | 0.001654348 |
| Cholinergic synapse | 16 | 0.00106 | 0.002255319 |
| Carbohydrate digestion and absorption | 6 | 0.00118 | 0.002458333 |
| Wnt signaling pathway | 21 | 0.00132 | 0.002693878 |
| Leukocyte transendothelial migration | 17 | 0.00162 | 0.00324 |
| Herpes simplex infection | 16 | 0.00254 | 0.004980392 |
| Prion diseases | 6 | 0.00286 | 0.0055 |
| Influenza A | 16 | 0.00377 | 0.007113208 |
| Apoptosis | 13 | 0.00584 | 0.01081481 |
| GnRH signaling pathway | 14 | 0.00679 | 0.01234545 |
| Cytokine-cytokine receptor interaction | 29 | 0.00757 | 0.01336842 |
| Inositol phosphate metabolism | 10 | 0.00762 | 0.01336842 |
| Aldosterone-regulated sodium reabsorption | 7 | 0.00931 | 0.01605172 |
| Leishmaniasis | 9 | 0.00956 | 0.01620339 |
| Oocyte meiosis | 15 | 0.00985 | 0.01641667 |
| Gap junction | 13 | 0.0105 | 0.01721311 |
| Adipocytokine signaling pathway | 10 | 0.0135 | 0.02177419 |
| ECM-receptor interaction | 12 | 0.0162 | 0.02571429 |
| Axon guidance | 15 | 0.0212 | 0.033125 |
| Maturity onset diabetes of the young | 5 | 0.0256 | 0.03938462 |
| Endocytosis | 13 | 0.028 | 0.04242424 |
| Cysteine and methionine metabolism | 6 | 0.0322 | 0.0480597 |
