# Supplementary material for: Circulating microRNAs in young individuals with long-duration type 1 diabetes in comparison with healthy controls
Source: Sci Rep. 2023 Jul 19;13:11634. doi: 10.1038/s41598-023-38615-7 (PMC10356803; doi:10.1038/s41598-023-38615-7)
Supplement: Supplementary file 2 — Supplementary Figure 1. [file 41598_2023_38615_MOESM2_ESM.pptx]

## Slide 1
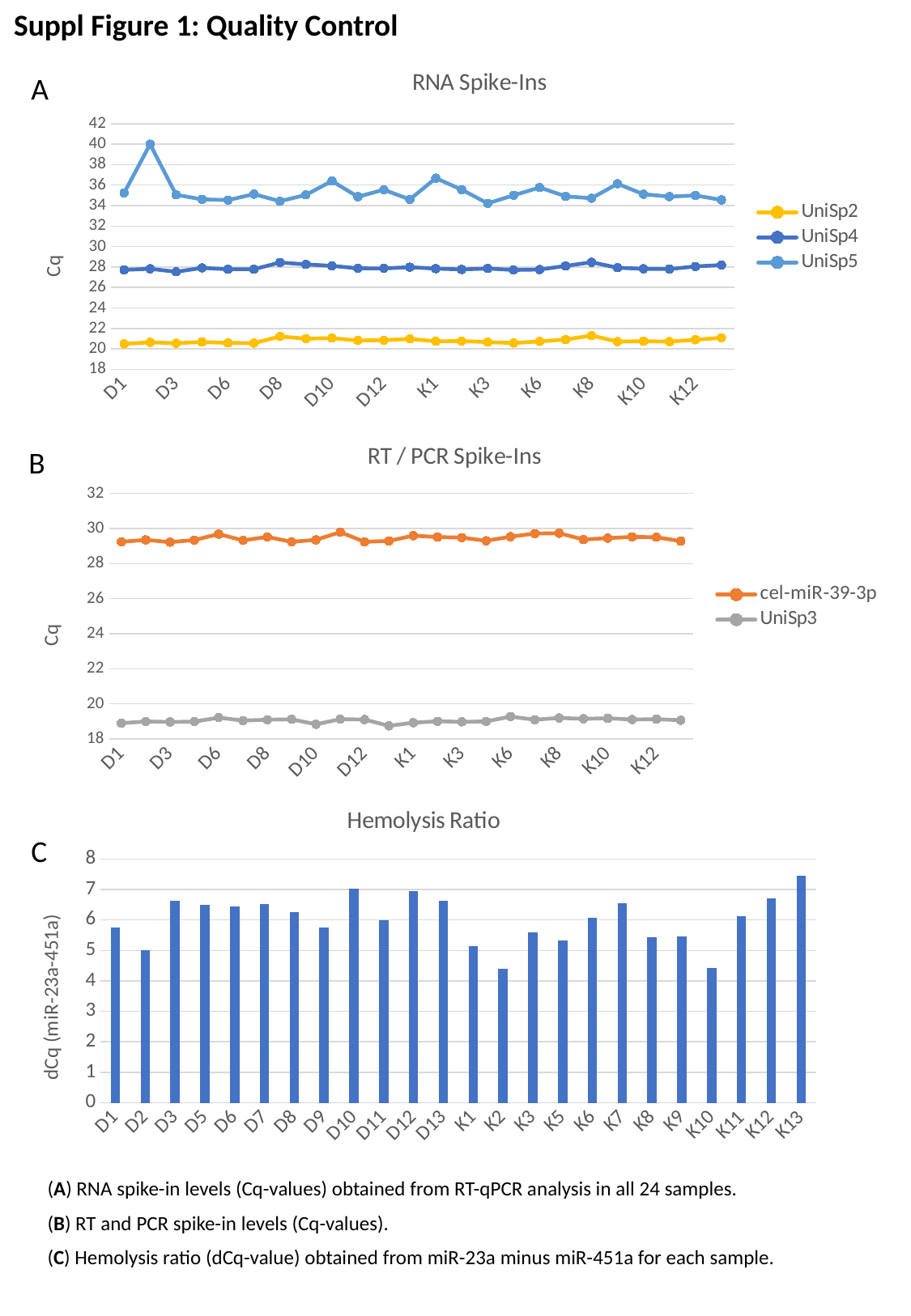

Suppl Figure 1: Quality Control
### Chart: RNA Spike-Ins
| Category | UniSp2 | UniSp4 | UniSp5 |
|---|---|---|---|
| D1 | 20.49 | 27.72 | 35.24 |
| D2 | 20.64 | 27.82 | 40.0 |
| D3 | 20.55 | 27.53 | 35.06 |
| D5 | 20.68 | 27.91 | 34.61 |
| D6 | 20.58 | 27.78 | 34.54 |
| D7 | 20.56 | 27.78 | 35.12 |
| D8 | 21.21 | 28.44 | 34.43 |
| D9 | 21.0 | 28.25 | 35.05 |
| D10 | 21.05 | 28.11 | 36.4 |
| D11 | 20.82 | 27.87 | 34.87 |
| D12 | 20.85 | 27.86 | 35.55 |
| D13 | 20.97 | 27.98 | 34.6 |
| K1 | 20.74 | 27.84 | 36.68 |
| K2 | 20.76 | 27.76 | 35.56 |
| K3 | 20.66 | 27.85 | 34.22 |
| K5 | 20.57 | 27.71 | 35.01 |
| K6 | 20.73 | 27.75 | 35.77 |
| K7 | 20.91 | 28.1 | 34.91 |
| K8 | 21.3 | 28.46 | 34.72 |
| K9 | 20.71 | 27.93 | 36.14 |
| K10 | 20.75 | 27.82 | 35.11 |
| K11 | 20.71 | 27.8 | 34.88 |
| K12 | 20.89 | 28.05 | 34.99 |
| K13 | 21.09 | 28.19 | 34.56 |A
### Chart: RT / PCR Spike-Ins
| Category | cel-miR-39-3p | UniSp3 |
|---|---|---|
| D1 | 29.24 | 18.9 |
| D2 | 29.35 | 19.0 |
| D3 | 29.22 | 18.97 |
| D5 | 29.34 | 18.99 |
| D6 | 29.68 | 19.22 |
| D7 | 29.32 | 19.05 |
| D8 | 29.52 | 19.1 |
| D9 | 29.24 | 19.12 |
| D10 | 29.35 | 18.84 |
| D11 | 29.79 | 19.13 |
| D12 | 29.24 | 19.11 |
| D13 | 29.29 | 18.75 |
| K1 | 29.59 | 18.93 |
| K2 | 29.51 | 19.01 |
| K3 | 29.48 | 18.98 |
| K5 | 29.3 | 19.0 |
| K6 | 29.53 | 19.28 |
| K7 | 29.71 | 19.1 |
| K8 | 29.73 | 19.2 |
| K9 | 29.37 | 19.15 |
| K10 | 29.45 | 19.18 |
| K11 | 29.52 | 19.11 |
| K12 | 29.5 | 19.13 |
| K13 | 29.28 | 19.07 |B
### Chart: Hemolysis Ratio
| Category | dCq miR-23a-451a |
|---|---|
| D1 | 5.75 |
| D2 | 5.0 |
| D3 | 6.640000000000001 |
| D5 | 6.489999999999998 |
| D6 | 6.43 |
| D7 | 6.509999999999998 |
| D8 | 6.259999999999998 |
| D9 | 5.75 |
| D10 | 7.02 |
| D11 | 5.979999999999997 |
| D12 | 6.949999999999999 |
| D13 | 6.620000000000001 |
| K1 | 5.140000000000001 |
| K2 | 4.399999999999999 |
| K3 | 5.579999999999998 |
| K5 | 5.32 |
| K6 | 6.07 |
| K7 | 6.559999999999999 |
| K8 | 5.420000000000002 |
| K9 | 5.469999999999999 |
| K10 | 4.43 |
| K11 | 6.120000000000001 |
| K12 | 6.719999999999999 |
| K13 | 7.440000000000001 |C
(A) RNA spike-in levels (Cq-values) obtained from RT-qPCR analysis in all 24 samples.
(B) RT and PCR spike-in levels (Cq-values).
(C) Hemolysis ratio (dCq-value) obtained from miR-23a minus miR-451a for each sample.
