# Supplementary material for: Circulating microRNAs in young individuals with long-duration type 1 diabetes in comparison with healthy controls
Source: Sci Rep. 2023 Jul 19;13:11634. doi: 10.1038/s41598-023-38615-7 (PMC10356803; doi:10.1038/s41598-023-38615-7)
Supplement: Supplementary file 3 — Supplementary Figure 2. [file 41598_2023_38615_MOESM3_ESM.pptx]

## Slide 1
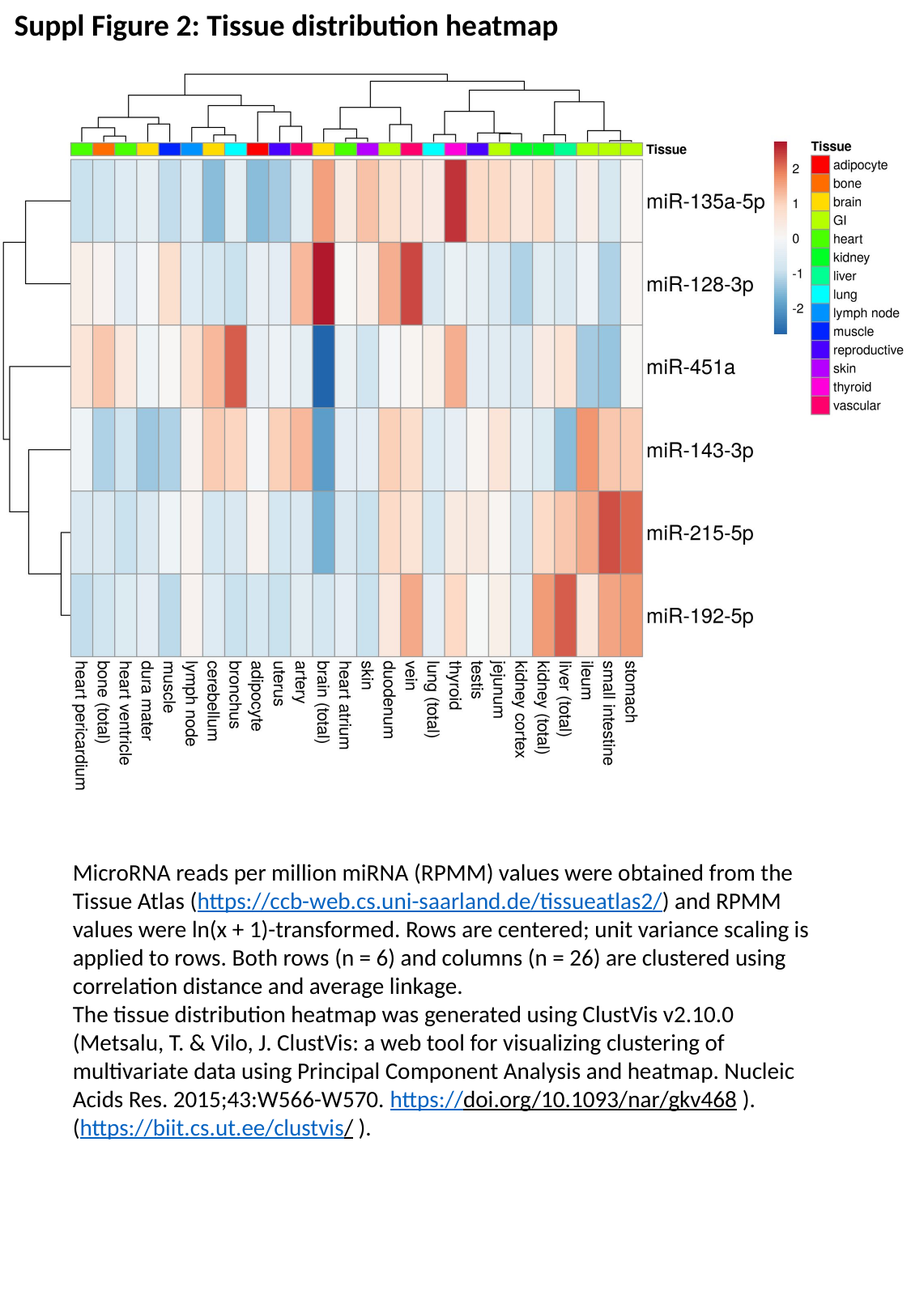

Suppl Figure 2: Tissue distribution heatmap
MicroRNA reads per million miRNA (RPMM) values were obtained from the Tissue Atlas (https://ccb-web.cs.uni-saarland.de/tissueatlas2/) and RPMM values were ln(x + 1)-transformed. Rows are centered; unit variance scaling is applied to rows. Both rows (n = 6) and columns (n = 26) are clustered using correlation distance and average linkage.
The tissue distribution heatmap was generated using ClustVis v2.10.0 (Metsalu, T. & Vilo, J. ClustVis: a web tool for visualizing clustering of multivariate data using Principal Component Analysis and heatmap. Nucleic Acids Res. 2015;43:W566-W570. https://doi.org/10.1093/nar/gkv468 ).
(https://biit.cs.ut.ee/clustvis/ ).
