# Supplementary material for: Circulating microRNAs in young individuals with long-duration type 1 diabetes in comparison with healthy controls
Source: Sci Rep. 2023 Jul 19;13:11634. doi: 10.1038/s41598-023-38615-7 (PMC10356803; doi:10.1038/s41598-023-38615-7)
Supplement: Supplementary file 4 — Supplementary Figure 3. [file 41598_2023_38615_MOESM4_ESM.pptx]

## Slide 1
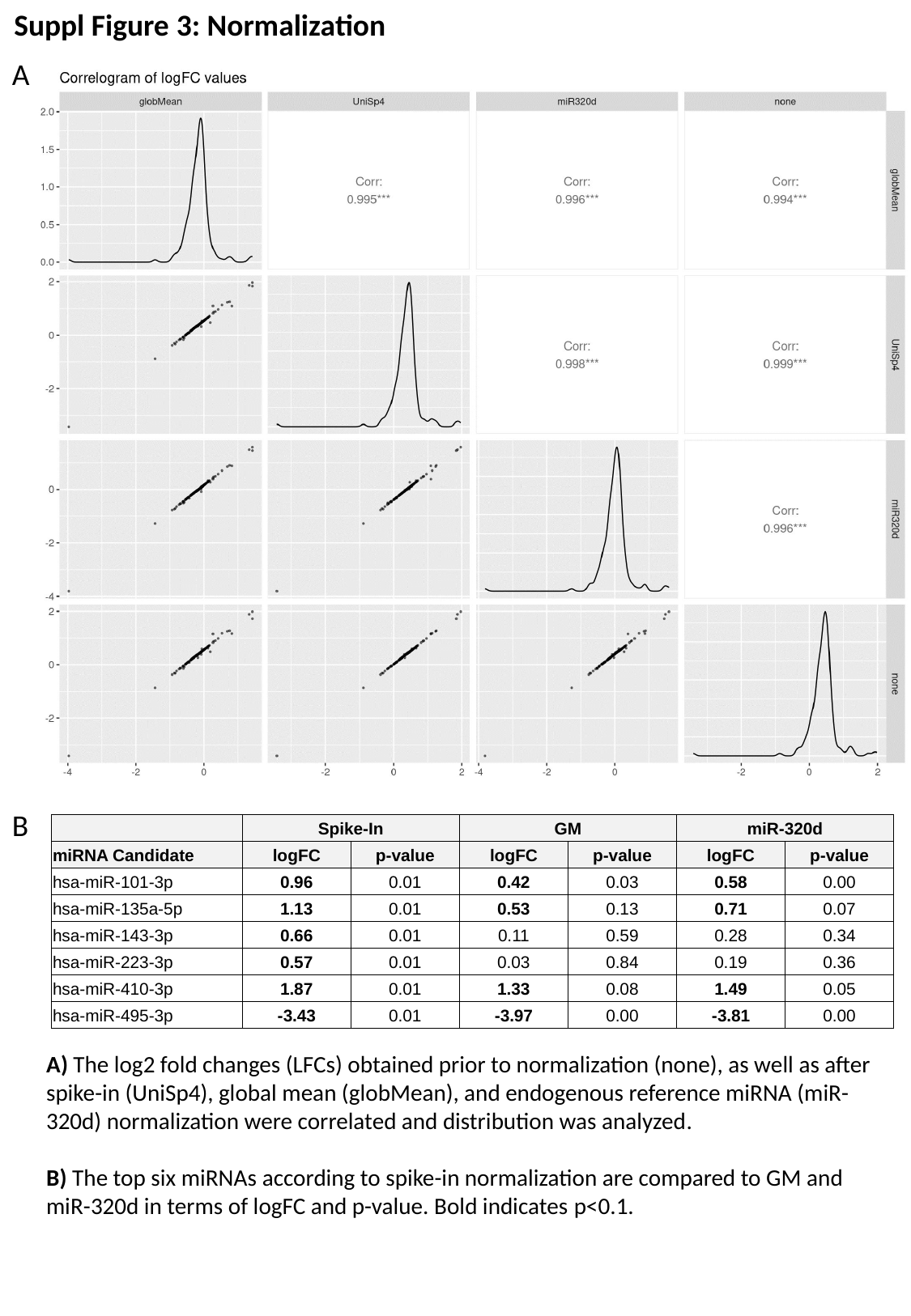

Suppl Figure 3: Normalization
A
B
| | Spike-In | | GM | | miR-320d | |
| --- | --- | --- | --- | --- | --- | --- |
| miRNA Candidate | logFC | p-value | logFC | p-value | logFC | p-value |
| hsa-miR-101-3p | 0.96 | 0.01 | 0.42 | 0.03 | 0.58 | 0.00 |
| hsa-miR-135a-5p | 1.13 | 0.01 | 0.53 | 0.13 | 0.71 | 0.07 |
| hsa-miR-143-3p | 0.66 | 0.01 | 0.11 | 0.59 | 0.28 | 0.34 |
| hsa-miR-223-3p | 0.57 | 0.01 | 0.03 | 0.84 | 0.19 | 0.36 |
| hsa-miR-410-3p | 1.87 | 0.01 | 1.33 | 0.08 | 1.49 | 0.05 |
| hsa-miR-495-3p | -3.43 | 0.01 | -3.97 | 0.00 | -3.81 | 0.00 |
A) The log2 fold changes (LFCs) obtained prior to normalization (none), as well as after spike-in (UniSp4), global mean (globMean), and endogenous reference miRNA (miR-320d) normalization were correlated and distribution was analyzed.
B) The top six miRNAs according to spike-in normalization are compared to GM and miR-320d in terms of logFC and p-value. Bold indicates p<0.1.
