# Supplementary material for: Circulating microRNAs in young individuals with long-duration type 1 diabetes in comparison with healthy controls
Source: Sci Rep. 2023 Jul 19;13:11634. doi: 10.1038/s41598-023-38615-7 (PMC10356803; doi:10.1038/s41598-023-38615-7)
Supplement: Supplementary file 5 — Supplementary Figure 4. [file 41598_2023_38615_MOESM5_ESM.pptx]

## Slide 1
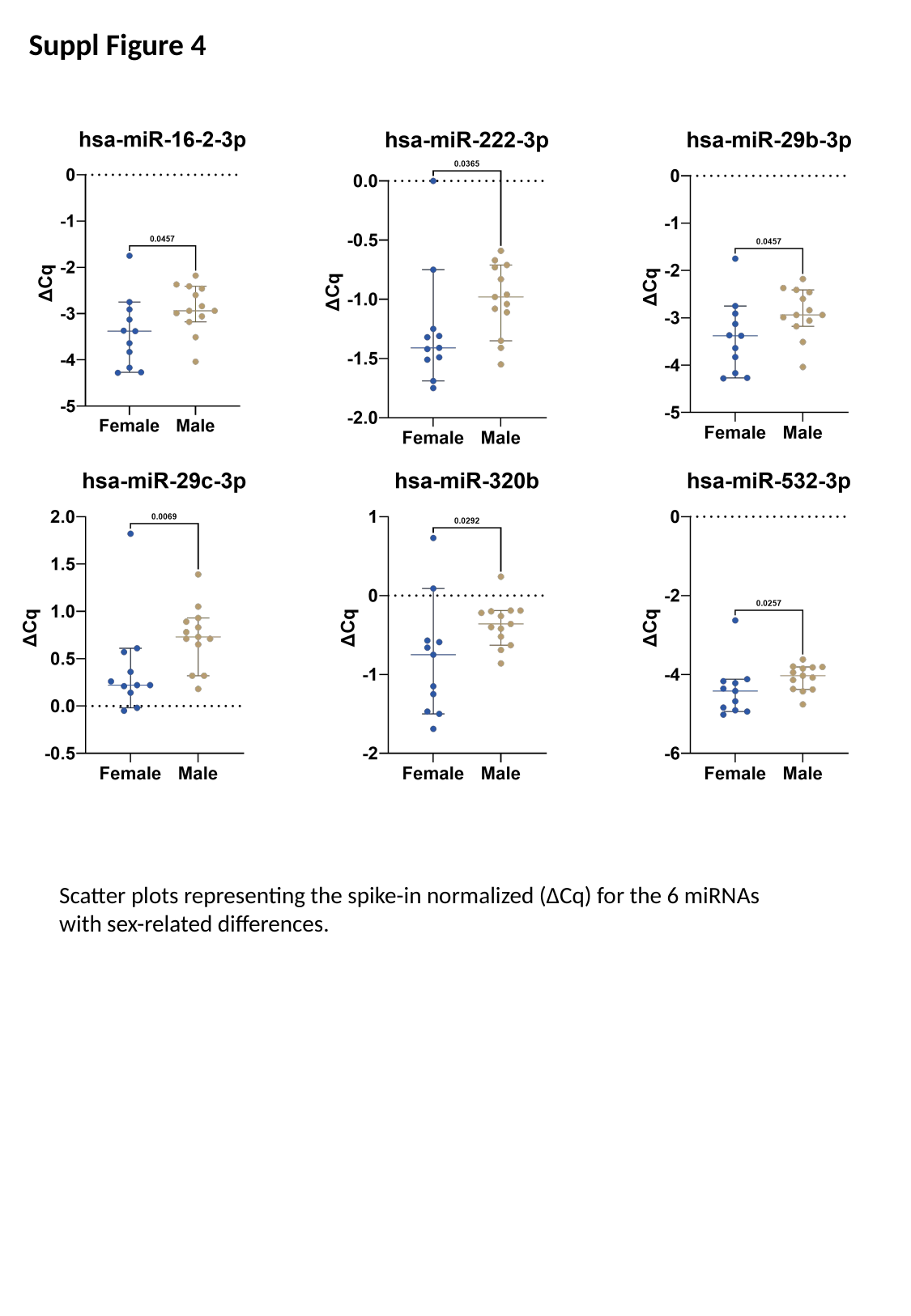

Suppl Figure 4
Scatter plots representing the spike-in normalized (ΔCq) for the 6 miRNAs with sex-related differences.
